# Endoscopic removal of a toothpick perforating the sigmoid colon and causing chronic abdominal pain: a case report

**DOI:** 10.4076/1757-1626-2-8469

**Published:** 2009-08-06

**Authors:** Petros Zezos, Anastasia Oikonomou, Vasilios Souftas, Dimitrios Gkotsis, Michail Pitiakoudis, Georgios Kouklakis

**Affiliations:** 1 Department of Gastroenterology and HepatologyMilitary General Hospital of Alexandroupolis, 68100 AlexandroupolisGreece; 2 Endoscopy UnitDemocritus University of Thrace, University General Hospital of Alexandroupolis, 68100 AlexandroupolisGreece; 3 Department of RadiologyDemocritus University of Thrace, University General Hospital of Alexandroupolis, 68100 AlexandroupolisGreece; 4 2^nd^ Department of SurgeryDemocritus University of Thrace, University General Hospital of Alexandroupolis, 68100 AlexandroupolisGreece

## Abstract

Toothpick ingestion is implicated in gut injuries which may cause severe complications, mimicking diseases causing acute abdomen. However, toothpick ingestion-related perforation may also cause mild, non-specific gastrointestinal symptoms without significant findings or major complications. We describe a young male with chronic postprandial lower abdominal pain caused by a toothpick impaction at the rectosigmoid junction after inadvertent ingestion. The foreign body was detected and successfully removed during flexible sigmoidoscopy. Perforation due to foreign body ingestion must be considered in the differential diagnosis in patients presenting with unexplained symptoms and findings, even when they do not recall any foreign body ingestion.

## Introduction

Foreign body (FB) ingestion is a common problem in everyday emergency clinical practice. It is well known that, fortunately, the majority of the ingested foreign objects pass the gastrointestinal (GI) tract spontaneously without complications. However, 10% to 20% require endoscopic removal and 1% or less requires surgical intervention [1].

In general, the navigation of an ingested foreign body depends on the anatomic conditions of the gastrointestinal tract (physiologic or pathologic) and on factors related to the ingested foreign body. Complications after foreign body ingestion (impaction, perforation, or obstruction) most often occur in areas of acute angulation or physiologic narrowing of the gastrointestinal tract, such as the level of the cricopharyngeus muscle and the ileocecal valve. Other less clinically significant locations are the level of the aortic arch and the left main stem bronchus in the esophagus, the gastroesophageal junction, the pylorus, the ligament of Treitz, the rectosigmoid junction and the anus. Moreover, patients with prior gastrointestinal tract surgery, congenital gut malformations, bowel strictures or obstructing conditions (inflammatory bowel disease, cancer, and diverticulosis) are at increased risk for obstruction or perforation. The majority of ingested foreign bodies which pass successfully through the esophagus will eventually pass through the entire GI tract uneventfully. However, the risk of perforation is higher when long, sharp or pointed metallic objects, animal or fish bones, or toothpicks are ingested [2,3].

## Case presentation

A 22-year-old Greek man was admitted to our hospital with an 18-month history of recurrent lower abdominal pain. The pain was located at the hypogastrium and intensified after the ingestion of a meal. The patient also complained about postprandial urgency for defecation which temporarily worsened the pain. Finally, the pain was totally relieved after defecation. He did not mention any other symptoms including fever, nausea, diarrhea, constipation, weight loss, rectal bleeding.

The patient stressed that his symptoms had begun a few days after the inadvertent ingestion of a toothpick, which was had been concealed in a fast-food potato by his friends for a joke. He reported that he bolted the potato without chewing, and although he was informed about the joke he never saw the toothpick in his stools. Shortly after that event, he suffered from postprandial abdominal pain and urgency for defecation. He sought for medical help at the emergency department of a city general hospital, but due to the negative results of the examinations, the symptoms were attributed to irritable bowel syndrome. Thereafter, the patient was accustomed to the postprandial abdominal pain without treatment. When he joined the army, progressive worsening of the severity and the frequency of the abdominal pain led him to the emergency department of our hospital.

His past medical history was unremarkable for significant illness including previous abdominal surgical procedures. The patient did not take any medications; he was a smoker (20 cigarettes per day) and consumed small amounts of alcohol infrequently (20 g once or twice a week). Physical examination revealed a nervous, healthy, well-nourished young man, with normal vital signs without any clinical findings except for a mild discomfort to deep palpation of the hypogastrium. The bowel sounds were normal. There were no palpable masses, no guarding or peritoneal signs. The rectal examination revealed normal sphincter tone, no tenderness or mass, and normal appearance of the stool. The laboratory data (complete blood count, chemistries, urinalysis, stools for heme, ova and parasites) and the plain abdominal X-ray were normal.

**Figure 1.**
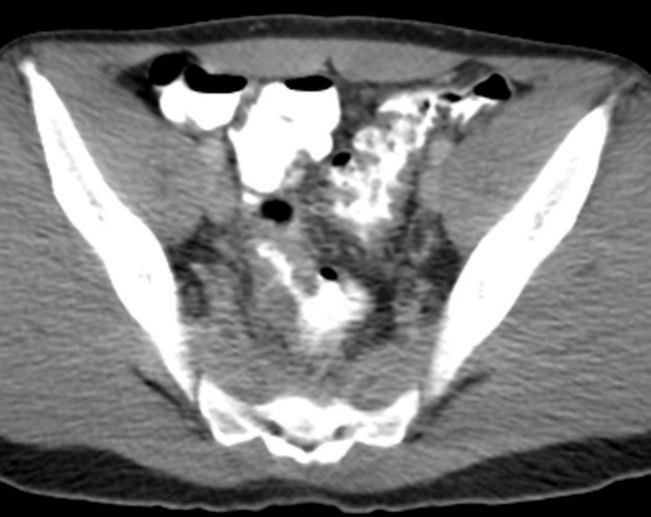
CT scan of the pelvis shows diffuse and “ulcer-like” severe thickening of the sigmoid wall with sparse diverticulas and perisigmoid fat infiltration.

Subsequently, a flexible sigmoidoscopy was performed and mucosal edema with erythema was noted at the rectosigmoid junction which was fixed with lumen narrowing that rendered the propulsion of the endoscope tip to the sigmoid colon quite difficult. No toothpick was noted at that time. Abdominal and pelvic computed tomography (CT) scan was performed subsequently and revealed severe segmental wall thickening, sparse diverticulas, pericolic fat infiltration and peritoneal thickening at the sigmoid colon region (Figure 1). No penetrating foreign body (toothpick) or pericolic abscess was detected.

**Figure 2.**
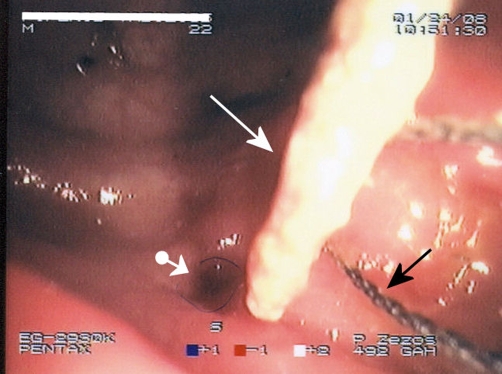
Endoscopic view of a toothpick impacted in the wall of sigmoid colon (white solid arrow), the small divot on the bowel wall opposite to the toothpick’s free end (white arrowhead-ball line) and the loop of a polypectomy snare before the capture and removal of the foreign body (black solid arrow).

**Figure 3.**
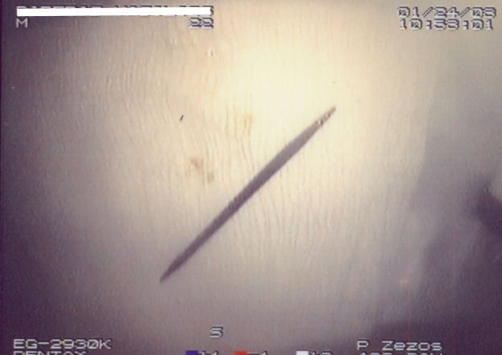
The wooden toothpick (6.5 cm × 0.3 cm) after the endoscopic removal from the sigmoid colon wall.

A second flexible sigmoidoscopy followed in order to obtain biopsies from the inflamed sigmoid colon and a toothpick was detected lodged in the rectosigmoid junction with the one end protruding freely into the bowel lumen and the other end impacted into the bowel wall (Figure 2). Additionally, erythematous mucosa and sparse tiny openings with whitish discharge were observed on the bowel wall opposite the toothpick’s free end (Figure 2). The free end of the toothpick was carefully and firmly grasped with a polypectomy snare (Figure 2) and the foreign body was gently dislodged from the colon wall. During the withdrawal of the endoscope the toothpick slipped from the snare loop into the distal rectum and the 6.5 by 0.3 cm wooden toothpick was finally easily removed intact through the anus by the examiner’s finger (Figure 3). The procedure was uneventful and the post-procedure abdominal X-ray was negative for pneumoperitoneum. Treatment with intravenous broad spectrum antibiotics was started while the patient remained fasted for 24 hours. A regular diet was instituted gradually during the next few days and intravenous antibiotics were switched to oral. During this period, the patient noted a complete remission of his postprandial abdominal pain and urgency for defecation. Finally, the patient was discharged from the hospital in excellent health and was given oral antibiotics for 5 more days.

**Figure 4.**
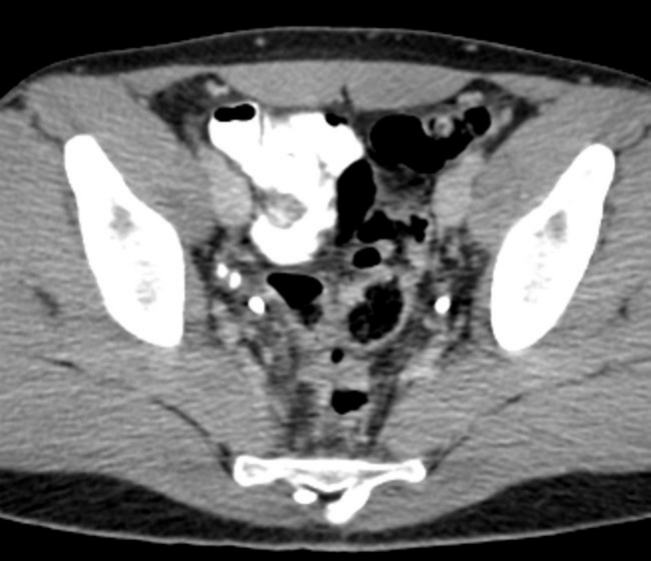
Follow-up CT scan of the pelvis, one month post-toothpick removal, shows significant improvement of sigmoid wall thickening and regression of pericolic fat stranding.

One month later the patient was completely asymptomatic and was re-admitted to the hospital for a scheduled follow-up. Abdominal and pelvic CT scan showed a dramatic improvement of the findings with decrease of sigmoid colon wall thickening and resolution of pericolic fat infiltration and peritoneal thickening (Figure 4). Colonoscopy up to the terminal ileum was normal except for mild segmental mucosal erythema and edema at the rectosigmoid junction. Colonic biopsies from the rectum and the sigmoid colon revealed mild chronic non-specific inflammatory reaction, without specific findings of Crohn’s or any other form of colitis.

## Discussion

Foreign body ingestion is a common clinical problem. The majority of the ingested foreign objects will pass the gastrointestinal tract spontaneously without complications. Selivanov et al. in a review of 10-year experience with 101 foreign body ingestions reported that if the ingested foreign bodies (FBs) reach the stomach, most of them (80%) will pass uneventfully through the entire GI tract [4]. Moreover, Velitchkov et al. in a retrospective analysis of 542 cases of ingested foreign bodies (esophageal FBs excluded) reported that 75.6% (410 out of 542) of FBs passed spontaneously. Endoscopic removal was successful in 106 cases of FB ingestion (19.6%) while in 26 cases (4.8%) surgical removal was required [5].

The vast majority of the literature support that the properties of the ingested foreign body determine the likelihood of complications including impaction, perforation, obstruction and bleeding. It is obvious that large, thin, and sharp objects (chicken or fish bones, metal objects, and wood splinters or toothpicks) carry a higher risk for gut perforation [5]. Gracia et al. reported that swallowed objects larger than 6.5 cm in length that passed beyond the gastro-esophageal junction were more prone to complications and required surgical intervention [6]. Furthermore, Goh et al. in a retrospective analysis of 62 patients treated for ingested FB perforation of the GI tract reported that 55 out of the 59 FBs (93%) recovered during surgery, were toothpicks, fish bones or bone fragments. Overall, toothpicks accounted for 8% (5 out of 62 cases) of the perforations [7].

In a 4-year survey in the United States, from 1979 to 1982, Budnick reported an estimated incidence of 3.6 toothpick-related injuries per 100,000 persons per year (approximately 8200 toothpick-related injuries yearly). Five percent of these injuries involved internal organs (0.2/100,000 persons) [8]. Toothpick ingestion is commonly implicated in gut injuries due to the bilateral sharply pointed ends and the length (approximately 6.5 cm) of this indigestible, hard foreign body, which causes difficulty in traversing the intestinal lumen, especially in the narrow or tortuous sections of the GI tract or at the transition from a mobile portion of the bowel (ileum and sigmoid) to a more fixed portion (cecum and rectum) [9]. Nevertheless, toothpick-related perforations have occurred throughout the GI tract, including the stomach, duodenum, small bowel, Meckel’s diverticulum, appendix, cecum, sigmoid colon, and rectum, with complications including abscesses, peritonitis, obstruction, hemorrhage, and perforations into adjacent organs or vessels resulting in severe morbidity or even death [10].

Recently, Li and Ender have reported in a review of 57 cases with toothpick ingestion-related gut injuries that the majority of patients did not recall the swallowing of a toothpick, while only 12% of patients remembered swallowing a toothpick, and another 21% of patients remembered eating food preparations containing toothpicks without swallowing the toothpick. In patients who remembered the ingestion of the toothpick the onset of symptoms ranged from less than a day to 15 years. The duration of symptoms before diagnosis ranged from 1 day to 9 months [11]. Apart from the significant morbidity, the toothpick ingestion was related with high mortality rate (18%) [11]. The most common site of injury was the duodenum (25%), followed by the sigmoid colon (14%). In about 20% (12/57) of cases, toothpicks migrated outside the GI tract penetrating into adjacent organs including the pleura, pericardium, peritoneum, ureter, bladder, aorta, inferior vena cava [11].

In the case we described some of the typical and common features of toothpick ingestion are missing. We presented a healthy young man with chronic, mild, recurrent lower abdominal pain that started a few days after the accidental ingestion of a toothpick. The patient had normal mental status, normal teeth, did not drink excessive amounts of alcohol and was aware of a toothpick ingestion 18 months ago. Despite seeking for medical help early on the occurrence of his symptoms, the inability to identify the foreign body at that time and the constant but non-specific mild clinical presentation delayed the diagnosis for 18 months. We speculate that our patient did not present with acute abdomen symptoms because the toothpick had caused a chronic, gradual perforation of the sigmoid colon wall [7]. Moreover, we also believe that the features of the abdominal pain which resembled those of the irritable bowel syndrome can be explained by the increased bowel peristalsis around the lodged toothpick during the normally released postprandial gastrocolic reflex [12].

The value of imaging studies for the diagnosis of ingested toothpicks is limited [11]. Plain abdominal X-ray and CT scan of the abdomen did not identify the radiolucent toothpick in our patient, but we consider the role of the latter equally crucial in determining the inflammatory reaction in and around the sigmoid wall, and in excluding findings requiring surgical intervention.

We successfully identified the foreign body during the second endoscopy and the endoscopic removal was performed without difficulties using a polypectomy snare since the toothpick was wedged into the sigmoid wall at one point with the other one projecting into the lumen. No post-procedural complications were noted and almost complete regression of the segmental bowel inflammation was observed one month later. The inflammatory narrowing of the sigmoid lumen and the small divots on the colonic wall oriented us to the possible site of FB impaction. We believe that the sparse diverticula in sigmoid colon observed in CT scan, correspond to the small divots in the colonic wall formed by the multiple and continuous penetrations of the free end of the toothpick that was protruding into the gut lumen.

Colonoscopic removal of toothpicks and other ingested foreign bodies obviating the need for surgical operation has been increasingly reported during the last two decades [13-16]. While surgical consultation is always needed, even when the foreign body perforation is chronic and uncomplicated, colonoscopy should be considered as the first step in management, since it is a potent and safe diagnostic and therapeutic tool in experienced hands. On the other hand, surgical treatment is mandatory in the presence of complications such as peritonitis, abscesses, fistulas, or FB migration to adjacent extra-colonic structures.

## Conclusion

Even though toothpicks are considered as relatively benign objects, there is increasing evidence in the literature that their ingestion may cause severe, even fatal, complications, mimicking acute abdomen. On the other hand, our case clearly illustrates that toothpick ingestion-related perforation may also cause mild, non-specific gastrointestinal symptoms without significant findings or major complications. Whichever the clinical presentation, acute or chronic, a perforation due to foreign body ingestion must be considered in the differential diagnosis in patients presenting with unexplained symptoms and findings, even when they do not recall any foreign body ingestion.

## Abbreviations

CT: Computed tomography
FB: Foreign body
GI: Gastrointestinal
